# Roles of Endomembrane Alkali Cation/Proton Exchangers in Synaptic Function and Neurodevelopmental Disorders

**DOI:** 10.3389/fphys.2022.892196

**Published:** 2022-04-25

**Authors:** Andy Y. L. Gao, Etienne Lourdin-De Filippis, John Orlowski, R. Anne McKinney

**Affiliations:** ^1^ Integrated Program in Neuroscience, McGill University, Montreal, QC, Canada; ^2^ Department of Pharmacology & Therapeutics, McGill University, Montreal, QC, Canada; ^3^ Graduate School of Engineering, Polytech Nice-Sophia, University of Nice Sophia, Antipolis, France; ^4^ Department of Physiology, McGill University, Montreal, QC, Canada

**Keywords:** endomembranes, alkali cation/proton exchangers, pH regulation, synaptic function, neurodevelopmental disorders, autism, attention deficit hyperactivity disorder, SLC9 gene family

## Abstract

Endomembrane alkali cation (Na^+^, K^+^)/proton (H^+^) exchangers (eNHEs) are increasingly associated with neurological disorders. These eNHEs play integral roles in regulating the luminal pH, processing, and trafficking of cargo along the secretory (Golgi and post-Golgi vesicles) and endocytic (early, recycling, and late endosomes) pathways, essential regulatory processes vital for neuronal development and plasticity. Given the complex morphology and compartmentalization of multipolar neurons, the contribution of eNHEs in maintaining optimal pH homeostasis and cargo trafficking is especially significant during periods of structural and functional development and remodeling. While the importance of eNHEs has been demonstrated in a variety of non-neuronal cell types, their involvement in neuronal function is less well understood. In this review, we will discuss their emerging roles in excitatory synaptic function, particularly as it pertains to cellular learning and remodeling. We will also explore their connections to neurodevelopmental conditions, including intellectual disability, autism, and attention deficit hyperactivity disorders.

## Introduction

In neurons, accurate execution of processes along the secretory and endolysosomal pathways is essential for the normal development, maturation, and remodeling of synaptic connections. For instance, glycosylation of newly synthesized neurotransmitter transporters (Melikian et al., 1996; Martinez-Maza et al., 2001; Li et al., 2004; Cai et al., 2005), neurotransmitter receptors (Jiang et al., 2006; Kaniakova et al., 2016; Sinitskiy et al., 2017), and voltage-gated ion channels (Zona et al., 1990; Ednie and Bennett, 2012; Baycin-Hizal et al., 2014; Scott and Panin, 2014) enhances their stability, membrane trafficking and/or activities that are crucial for membrane excitability, neurotransmission, and ultimately learning and memory (Matthies et al., 1999; Inaba et al., 2016). Likewise, optimal performance of the endolysosomal system is vital for neurotrophin signaling, presynaptic vesicle recycling, axonal growth cone migration, and synaptic plasticity (Morgan et al., 2013; Bowen et al., 2017; Terenzio et al., 2017; Hiester et al., 2018; Kiral et al., 2018; Naslavsky and Caplan, 2018). Unlike other cells in the body, the morphologies of neurons are quite elaborate, which creates additional complexity in the compartmentalization and regulation of protein trafficking between their somata, dendrites, and axons. In recent years, there has been increasing awareness that disruptions in biosynthetic and endolysosomal functions underlie several neurodevelopmental and neurodegenerative disorders involving synaptic deficits, eventually leading to cognitive impairments (Lefebvre et al., 2003; Aronica et al., 2005; Nixon, 2005; Baloyannis, 2014; Maxfield, 2014; Schreij et al., 2016; Wang et al., 2016; Bingol, 2018; Winckler et al., 2018). The underlying factors are assuredly multifactorial and complex. While significant advances have been made in identifying many of these factors, a detailed appreciation of how they cause neuronal dysfunction has yet to be fully realized. Increasing evidence has revealed a central role for pH homeostasis of endomembrane compartments in central nervous system function and neurological disorders (Jentsch and Pusch, 2018; Sou et al., 2019; Collins and Forgac, 2020; Khosrowabadi and Kellokumpu, 2020; Prasad and Rao, 2020). For the purposes of this review, we will highlight the importance of the endosomal system to excitatory synaptic plasticity in the brain, and then focus our discussion on the significant contributions of alkali cation (Na^+^ or K^+^)/proton (H^+^) exchangers (NHEs) (also referred to as the SLC9 gene family) to this process.

## Endosomal Trafficking in Neuronal Plasticity

**FIGURE 1:**
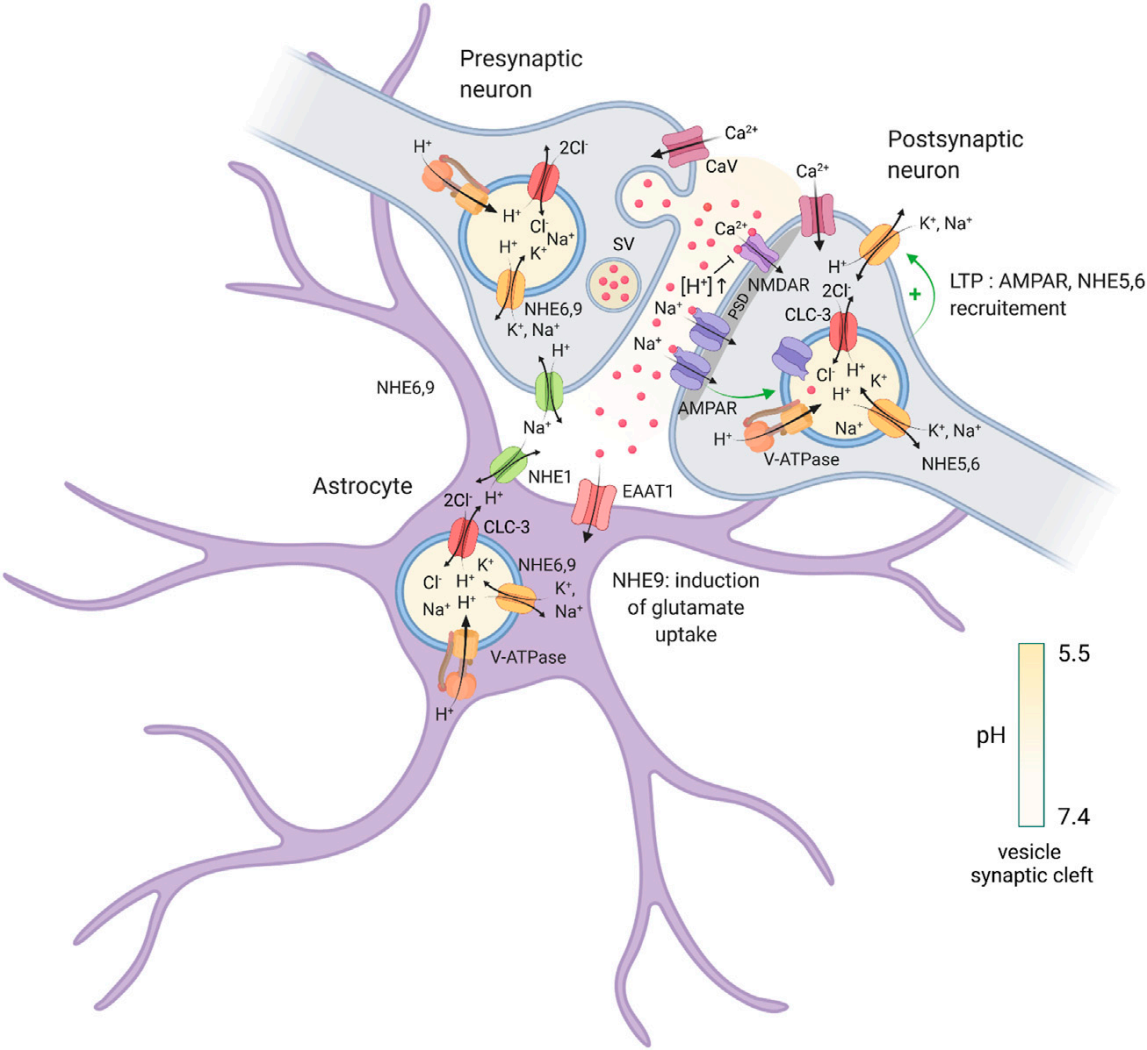
Regulation of intracellular pH at a tripartite synapse. Illustration of a tripartite synapse (presynaptic axon terminal, postsynaptic dendritic spine and astrocyte) and localization of different proteins involved in cytosolic and endosomal pH regulation and neurotransmission. V-ATPase, vacuolar H^+^-ATPase; NHE, (Na^+^, K^+^)/H^+^ exchangers; CLC-3, 2Cl^−^/H^+^ exchanger isoform 3; CaV, voltage-gated calcium channel; EAAT1, excitatory amino acid transporter 1; NMDAR, *N*-methyl-D-aspartate receptor; AMPAR, α-amino-3-hydroxy-5-methyl-4-isoxazolepropionic acid receptor; PSD, postsynaptic density; SV, synaptic vesicle; red balls; glutamate. Created with BioRender.com.

Studies investigating cognitive deficits commonly focus on the hippocampus, a medial temporal lobe structure that has long been implicated in learning and memory (Scoville and Milner, 1957; Penfield and Milner, 1958; Stuchlik, 2014). Pyramidal neurons in the hippocampus are dotted by dendritic spines, small (1 µm) heterogenous protrusions that emanate from the dendritic shaft and serve as the sites of excitatory postsynaptic connections (Gray, 1959; Bourne and Harris, 2008; McKinney, 2010). In general, dendritic spines contain a “head” structure joined to the dendritic shaft by a thinner “neck” apparatus (Harris et al., 1992). Under electron microscopy, the heads of dendritic spines contain an electron-dense region known as the postsynaptic density (PSD), which lies in direct apposition to the presynaptic active zone (Lei et al., 2016; Borovac et al., 2018) (Figure 1). The PSD typically contains hundreds of proteins involved in excitatory postsynaptic signaling, including ionotropic glutamatergic α-amino-3-hydroxy-5-methyl-4-isoxazolepropionic acid receptors (AMPARs) and *N*-methyl-D-aspartate receptors (NMDARs). AMPARs are non-specific cation channels that primarily conduct Na^+^ and K^+^ ions and are responsible for propagating fast excitatory neurotransmission. NMDARs are also permeable to monovalent cations but also conduct divalent ions such as Ca^2+^ which can activate downstream signaling cascades that alter synaptic strength (McKinney, 2010). As larger spines typically contain a larger PSD and more AMPARs, it is widely believed that spine size correlates with the strength of the corresponding synapse (Arellano et al., 2007). Spines are extremely dynamic structures that can alter in size following the induction of long-term potentiation (LTP) and long-term depression (LTD), cellular correlates of learning and memory. *In vitro* measurements of cultured hippocampal neurons initially revealed that bidirectional changes in spine morphology are associated with changes in synaptic strength. In particular, spine growth in response to LTP and shrinkage following LTD are believed to correspond to synaptic strengthening and weakening, respectively (Yuste and Bonhoeffer, 2004; Matsuzaki et al., 2004; Okamoto et al., 2004; Zhou et al., 2004; Holtmaat and Svoboda, 2009).

While extensive details into the mechanisms governing LTP and LTD are outside of the scope of this review, they are highly dependent upon the proper regulation of protein trafficking *via* endosomes (Henley and Wilkinson, 2013; Hiester et al., 2018; Parkinson and Hanley, 2018). This is perhaps best illustrated at the synapses between pyramidal cells in hippocampal areas *cornu ammonis* 3 (CA3) and CA1, which have been extensively utilized as experimental models to investigate cellular mechanisms of LTP and LTD. Importantly, LTP is dependent upon strong NMDAR activation following membrane depolarization in postsynaptic CA1 pyramidal neurons, which allows Ca^2+^ influx into the postsynaptic cell and the activation of various downstream kinases and mediators (Malenka and Nicoll, 1993; Malenka, 1994; Huganir and Nicoll, 2013). In the earlier stages of LTP, these mechanisms enable excitatory synaptic strengthening through an enhancement of AMPAR function, which may arise from changes in AMPAR properties or from the recruitment of additional AMPARs to postsynaptic sites (Isaac et al., 1995; Liao et al., 1995; Roche et al., 1996; Barria et al., 1997; Benke et al., 1998; Shi et al., 1999; Banke et al., 2000; Diering and Huganir, 2018). AMPARs usually assemble as tetramers of two homodimers out of four possible subunits (GluA1-4). It is generally believed that GluA1-containing AMPARs (i.e., GluA1/GluA2 heteromers or GluA1 homomers) are the principal population of receptors that initially traffic to excitatory synapses during LTP induction, thereby underlying the immediate potentiation in synaptic strength (Shi et al., 1999; Heynen et al., 2000; Broutman and Baudry, 2001; Lu et al., 2001). Dendritic spines also expand significantly in volume following LTP to allow for the addition of AMPARs to the PSD, thus serving as a morphological correlate for synaptic strengthening (Bourne and Harris, 2007; Bourne and Harris, 2008). Importantly, these events result from the exocytosis of recycling endosomes to the postsynaptic membrane, the disruption of which impairs activity-dependent spine growth (Park et al., 2004; Park et al., 2006; Kopec et al., 2007). Endosomes thus act as a source of both additional AMPARs and lipid membranes and can mediate both functional and structural enhancements of dendritic spines. Hence, it is logical to assume that disruptions in endosomal dynamics can deleteriously impact excitatory postsynaptic remodeling at CA3-CA1 synapses.

## Endomembrane pH Homeostasis

**FIGURE 2:**
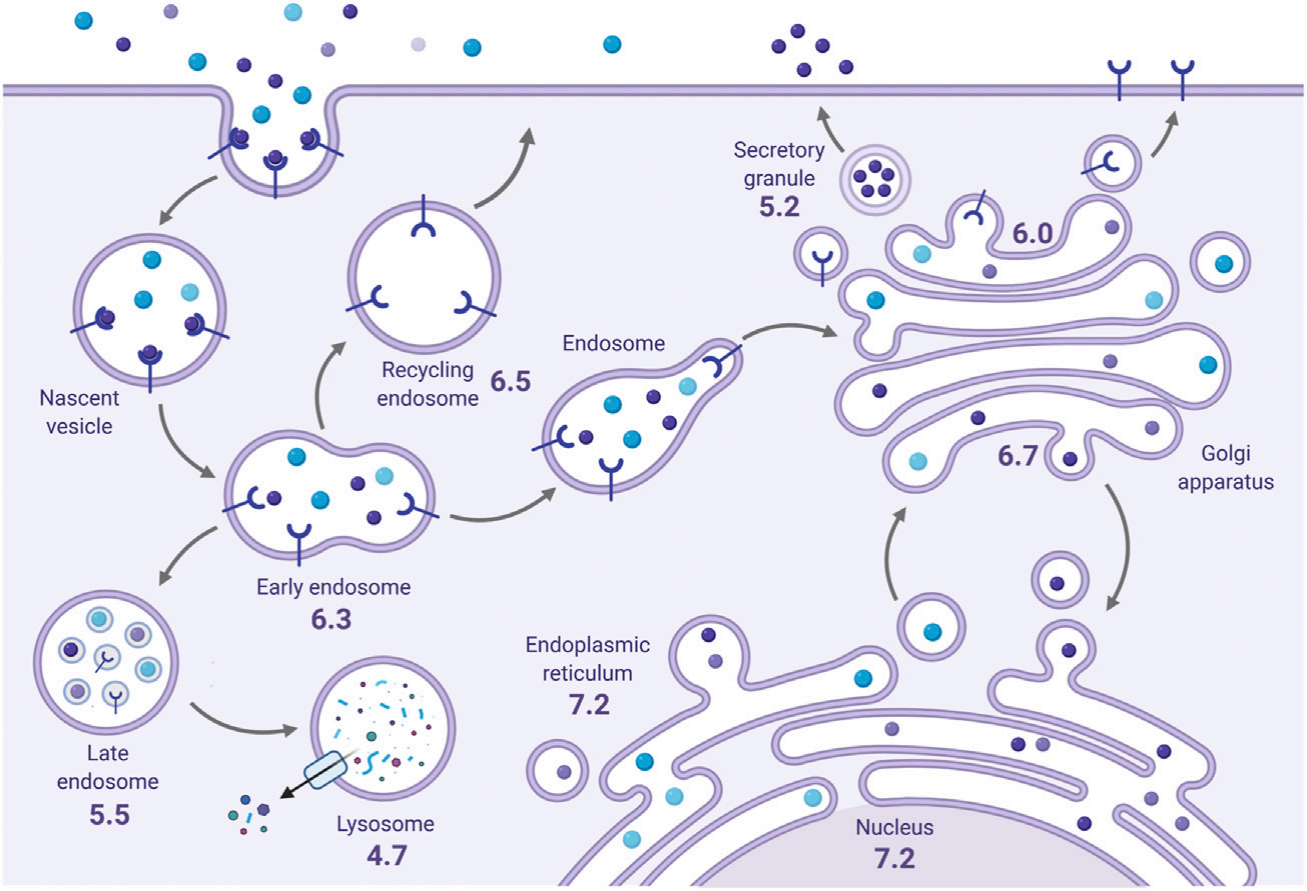
The steady-state pH of endomembrane compartments. The average resting pH of endomembrane compartments (indicated) becomes progressively more acidic along the secretory and endocytic pathways. Created with BioRender.com.

The internal pH of exocytic and endocytic compartments is an important determinant of their function and dynamics (Casey et al., 2010). For instance, the pH milieu of the endoplasmic reticulum (ER) is near neutral (pH ∼7.2) but becomes increasing acidic from the *cis-*Golgi network (pH ∼6.7) through to the *trans*-Golgi network (pH ∼6.0) (Figure 2). Secretory vesicles can attain even higher H^+^ concentrations (pH ∼5.2). Acidification of compartments along the biosynthetic pathway is important for proper post-translational processing, sorting and transport of newly synthesized proteins and lipids. Likewise, endocytosed membrane-bound proteins from the cell surface that are targeted for lysosomal degradation are subjected to a gradient of acidification from early endosomes (EE, pH ∼6.3) to late endosomes (LE, pH ∼5.5) and, finally, lysosomes (pH ∼4.7). Conversely, internalized cell-surface proteins destined for transport back to the plasma membrane may be trafficked through more alkaline recycling endosomes (RE, pH ∼6.5). Endolysosomal pH is involved in a number of functions, including 1) cargo sorting, 2) receptor-ligand dissociation and processing, 3) the maturation and transport of endolysosomal vesicles, 4) membrane protein and receptor recycling, and 5) the activity of luminal enzymes including degradative hydrolases (Casey et al., 2010; Hu et al., 2015). The proper regulation of luminal pH is thus vital for establishing the identity and function of each compartment. Vesicular acidification in both the secretory and endolysosomal pathways is primarily achieved by the vacuolar H^+^-ATPase (V-ATPase), an evolutionarily conserved multisubunit complex that utilizes the chemical energy present in ATP to pump H^+^ from the cytosol into the endomembrane lumen (Casey et al., 2010; Collins and Forgac, 2020; Vasanthakumar and Rubinstein, 2020). Because V-ATPase-mediated activity transports only H^+^ independently of other ions, this results in the generation of an inside-positive voltage gradient across organellar membranes that gradually impedes additional H^+^ influx, limiting acidification. However, this effect is offset by counterion conductances (anion influx or cation efflux). The most prominent counterion conductance is mediated by members of the CLC family of voltage-gated chloride channels and transporters, specifically CLC3-7, which localize to secretory and endolysosomal compartments and facilitate the entry of negatively-charged Cl^−^ anions to neutralize the membrane potential and allow for progressive acidification (Gentzsch et al., 2003; Guzman et al., 2015; Jentsch and Pusch, 2018). Notably, these CLC family members act as electrogenic 2Cl^−^/1H^+^ exchangers and can thus serve as both a mechanism of compensatory anion influx as well as H^+^ efflux (Picollo and Pusch, 2005; Scheel et al., 2005; Guzman et al., 2013; Rohrbough et al., 2018). However, CLCs are not the sole source of H^+^ efflux, as a significant fraction of this H^+^ leakage is also attributed to endomembrane members of the (Na^+^ or K^+^)/H^+^ exchanger (NHE) gene family (Nakamura et al., 2005; Orlowski and Grinstein, 2007).

## The NHE Family

NHEs are a family of evolutionarily conserved secondary active transporters that couple the countertransport of monovalent alkali cations (such as Na^+^, K^+^, or Li^+^) for H^+^ across biological membranes. To date, 13 distinct mammalian genes from three phylogenetically distinct categories have been identified, including NHE1 through NHE9 (encoded by the *SLC9A1-SLC9A9* genes), Na^+^/H^+^ antiporters 1 and 2 (NHA1-2, encoded by the *SLC9B1-2* genes), and the recently characterized NHE10 and NHE11 (*SLC9C1* and *SLC9C2* genes, respectively) (Brett et al., 2005a; Orlowski and Grinstein, 2011; Fuster and Alexander, 2014; Pedersen and Counillon, 2019). NHE proteins differ in their sequence length, drug sensitivity, cation selectivity, and tissue and subcellular localization, with some being predominantly localized to specific subdomains of the cell surface and others to intracellular organelles. Amongst the SLC9A isoforms, five (NHE1 through NHE5) are primarily active at the plasma membrane, while the remaining four (NHE6 through NHE9) localize mainly in intracellular compartments. Plasmalemmal NHEs mediate the electroneutral exchange of extracellular Na^+^ (or Li^+^) for cytosolic H^+^ (stoichiometry of 1:1 or 2:2) (Aronson, 1985; Fuster et al., 2008) and are mainly responsible for regulating systemic and cytosolic pH. In contrast, endomembrane NHEs mediate the electroneutral transport of luminal H^+^ for a cytosolic cation (such as Na^+^ or K^+^, with the latter being more prominent given its higher intracellular concentration) to alkalinize organellar pH (Numata and Orlowski, 2001; Brett et al., 2005b; Nakamura et al., 2005). Together, the NHE/SLC9A transporters regulate intracellular and organellar pH, volume, and osmolality, which in turn influences vesicular trafficking, signaling, cell growth and migration. The functions of the SLC9B and SLC9C isoforms are less well characterized. SLC9B isoforms are broadly expressed and have been implicated in hypertension (Xiang et al., 2007; Chintapalli et al., 2015; Kondapalli et al., 2017), insulin secretion (Deisl et al., 2013; Deisl et al., 2016) and sperm motility and fertility (Chen et al., 2016). By contrast, expression of the SLC9C isoforms is largely confined to testis where they are also essential for sperm function (Wang et al., 2003; Quill et al., 2006; Wang et al., 2007; Windler et al., 2018; Cavarocchi et al., 2021).

While NHEs are typically categorized as acting at the plasma membrane or endomembrane compartments, many of them shuttle continuously between cellular locations in a cell- or stimulus-dependent manner. Most notably, upon exit from the ER, the endomembrane NHEs (eNHEs) are trafficked through the secretory pathway and transiently appear at the cell surface prior to being internalized to their respective compartments (Orlowski and Grinstein, 2007). Moreover, although the phylogenetically-related epithelial-enriched NHE3 and neural-enriched NHE5 isoforms are often categorized as cell surface NHEs, they are also found within a pool of intracellular vesicles that rapidly shuttle to and from the plasma membrane in response to diverse stimuli (D’Souza et al., 1998; Janecki et al., 1998; Kurashima et al., 1998; Szászi et al., 2002; Yang et al., 2002; Donowitz et al., 2009; Bobulescu et al., 2010; Diering et al., 2011; Lukashova et al., 2011; Lukashova et al., 2013; Jinadasa et al., 2014). Indeed, the tissue expression and spatiotemporal membrane trafficking of these NHEs is a significant determinant of their function. For instance, NHE3 is present at the apical surface as well as intracellular vesicles of renal and gastrointestinal epithelia and plays critical roles in regulating not only ion secretion and resorption, indirectly mediating the transmembrane movement of fluid, but also regulating the pH of newly formed endosomes and internalization of select cargo (D’Souza et al., 1998; Gekle et al., 2001; Gekle et al., 2004; Orlowski and Grinstein, 2011; Fuster and Alexander, 2014; Pedersen and Counillon, 2019). Similarly, NHE5 has been detected in endosomes of C6 glioma cells that partially overlap markers for recycling endosomes where it has been implicated in modulating endosomal pH as well as cell surface delivery and signalling of the hepatocyte growth factor receptor (HGFR; also called the MET receptor tyrosine kinase) and epidermal growth factor receptor (Fan et al., 2016; Kurata et al., 2019). By contrast, other isoforms such as NHE6, NHE7, NHE8, and NHE9 are widely expressed where they reside mainly in intracellular compartments and, to a limited extent, at the plasma membrane (Nakamura et al., 2005; Orlowski and Grinstein, 2007; Ohgaki et al., 2011). Though these isoforms primarily regulate endomembrane pH homeostasis and trafficking, they can also be active at the cell surface of specialized cells (Goyal et al., 2005; Hill et al., 2006; Zhang et al., 2007; Xu et al., 2008).

## Roles of NHEs in the Central Nervous System

Increasing attention has recently been paid to the function of NHEs in brain. Earlier experiments investigating NHEs in excitatory synaptic plasticity showed that NHE blockade using the broad-spectrum inhibitor 5-(N-ethyl-N-isopropyl)-amiloride (EIPA) was found to significantly improve LTP maintenance in rat hippocampal slices (Ronicke et al., 2009). While the specific NHE isoform(s) responsible for this phenomenon was not determined, it is evident that NHE-mediated pH modulation can play a critical role in synaptic transmission and remodeling. Accordingly, disruption of some of these isoforms has been associated with neuropathologic conditions. Ablation of NHE1 in mice leads to a severe neurodegenerative phenotype associated with ataxia, seizures, and postnatal lethality (Cox et al., 1997; Bell et al., 1999). NHE1 has since been shown to regulate the release of the major inhibitory neurotransmitter γ-aminobutyric acid (GABA) (Jang et al., 2006; Dietrich and Morad, 2010; Bocker et al., 2019) and may thus play a larger role in mediating cellular excitability. Likewise, mutations in human NHE1 cause Lichtenstein-Knorr syndrome, an autosomal recessive disorder characterized by hearing loss and cerebellar ataxia (Guissart et al., 2015). Disruption of human NHE1 function has also been associated with other forms of ataxia, spastic paraplegia, intellectual disability, and epilepsy (Zhu et al., 2015; Iwama et al., 2018; Mendoza-Ferreira et al., 2018). Moreover, genetic variants in the human NHE7 have recently been shown to cause a non-syndromic form of intellectual disability accompanied by macrocephaly, minimal speech, muscular weakness and hypotonia (Khayat et al., 2019). NHE7 resides within the Golgi complex, accumulating predominantly in the *trans*-Golgi network and post-Golgi vesicles (Numata and Orlowski, 2001; Lin et al., 2005) where it regulates luminal pH as well as glycosylation of exported cargos (Khayat et al., 2019). Although further insights into the neuronal function of NHE7 are presently lacking, these findings illuminate the role of organellar pH in regulating learning and cognition in the brain.

The roles of two other closely-related (58% amino acid identity) eNHEs, NHE6, and NHE9, have also received considerable attention. Both eNHEs are expressed ubiquitously, though NHE6 is particularly enriched in brain (see https://www.ncbi.nlm.nih.gov/gene/10479 for *SLC9A6* and https://www.ncbi.nlm.nih.gov/gene/285195 for *SLC9A9*). These eNHEs localize predominantly to discrete but overlapping pools of EEs and REs (Nakamura et al., 2005; Orlowski and Grinstein, 2007; Deane et al., 2013) (Figure 1). In the human genome, the *SLC9A6/NHE6* and *SLC9A9/NHE9* genes localize at chromosomal positions Xq26.3 and 3q24, respectively. In humans, several mRNA splice-variants of NHE6 have been documented in research databases (https://www.ncbi.nlm.nih.gov/gene/10479). Thus far, only two of these, NHE6v1 and NHE6v2 (originally called NHE6.1 and NHE6.0, respectively) have been examined at the protein level, with the former containing an additional 32 amino acid insertion within its second extracellular loop. This insert contains an additional glycosylation site, though its functional significance, if any, remains uncertain. As expected, overexpression of NHE6 (Ohgaki et al., 2010; Xinhan et al., 2011; Ilie et al., 2016) and NHE9 (Kondapalli et al., 2013) in cultured cells resulted in the elevation of endosomal pH, whereas knock-down or ablation had the opposite effect (Ohgaki et al., 2010; Xinhan et al., 2011; Ouyang et al., 2013; Ullman et al., 2018). Most notably, deleterious mutations in these eNHEs have been directly associated with neurodevelopmental disorders. Mutations in NHE6 result in Christianson syndrome (CS), a monogenic disorder causing severe X-linked intellectual disability (XLID), non-verbalism, epilepsy, truncal ataxia, sleep disturbances, postnatal microcephaly, and autistic traits (Christianson et al., 1999; Gilfillan et al., 2008; Tarpey et al., 2009; Garbern et al., 2010; Schroer et al., 2010; Mignot et al., 2013; Ilie et al., 2019; Gruber et al., 2022). While CS is considered to be a rare disorder, *SLC9A6* is one of the six most commonly mutated loci in patients with XLID (Pescosolido et al., 2014), suggesting that mutations in this gene may be more common than previously thought. By contrast, variants in *SLC9A9/NHE9* are associated with attention deficit hyperactive disorder (ADHD) (de Silva et al., 2003; Lasky-Su et al., 2008) and autism spectrum disorder with epilepsy (ASD) (Morrow et al., 2008). Curiously, findings from post-mortem tissue of individuals diagnosed with idiopathic ASD have suggested a downregulation of NHE6 expression with a concomitant increase in that of NHE9 (Schwede et al., 2013), suggesting a possible reciprocity in the function of these two eNHEs in the development of ASD. Indeed, genes encoding endosomal regulators are highly dominant among risk genes for ASD, illustrating the importance of this system and, by extension, these eNHEs in the function of neurons and synapses (Patak et al., 2016). The mechanisms by which genetic disruptions in eNHEs result in such severe disorders of the nervous system are poorly understood and remain to be elucidated.

## Roles of NHEs in Vesicular Trafficking

Given their subcellular locations, it is perhaps not surprising that these eNHEs would play critical roles in endocytic cargo trafficking. Even in non-neuronal cells, one of the first physiological roles identified for NHE6 function was in the development of cellular polarity. In liver hepatoma HepG2 cells, which possess distinct apical and basolateral surface domains, knock-down of NHE6 selectively decreased endosomal pH and disrupted transcytotic recycling of proteins to the apical domain, thereby disrupting the development of proper cellular polarity (Ohgaki et al., 2010). NHE6 is further implicated in clathrin/adaptor protein 2 (AP2)-mediated endocytosis (CME) of cell-surface proteins. In HeLa cells, NHE6 colocalizes strongly with both clathrin and transferrin, a cellular iron carrier that is classically used to study CME into recycling endosomes (Xinhan et al., 2011; Ilie et al., 2016). Accordingly, knock-down of NHE6 attenuated transferrin uptake in HeLa cells, which was indicative of a role for NHE6 in trafficking of recycling endosomal cargo. However, manipulation of NHE6 expression did not impair CME of the activated epidermal growth factor, suggesting that NHE6 regulates only a subset of cargo internalized by CME (Xinhan et al., 2011; Ilie et al., 2016). NHE9 similarly colocalizes with transferrin in mouse primary cortical astrocytes, in which overexpressing NHE9 significantly enhanced transferrin uptake (Kondapalli et al., 2013). Interestingly, siRNA knockdown of NHE9 did not alter transferrin uptake, suggesting some degree of compensation from NHE6 in the absence of NHE9. Parenthetically, a recent report showed that NHE9 is present within microvascular endothelial cells that line the blood-brain barrier and mediates iron delivery to the brain (Beydoun et al., 2017). In response to iron starvation, NHE9 levels are upregulated, alkalinizing endosomal pH and promoting the recycling of transferrin receptors back to the cell surface to facilitate additional iron uptake (Beydoun et al., 2017). The importance of NHE6 and NHE9 in regulating transferrin uptake and CME in general may thus be context- and cell-type specific. It should be noted that in neurons, AMPARs, NMDARs, ionotropic GABA_A_ receptors, and other cell-surface proteins are predominantly internalized *via* CME (Kittler et al., 2000; Hanley, 2018). Therefore, alterations in eNHE function are predicted to perturb CME mechanisms in neurons and disrupt the regulation of neurotransmission and remodeling of both excitatory and inhibitory synapses.

## Roles of Endosomal NHEs in Neurodevelopment

**FIGURE 3:**
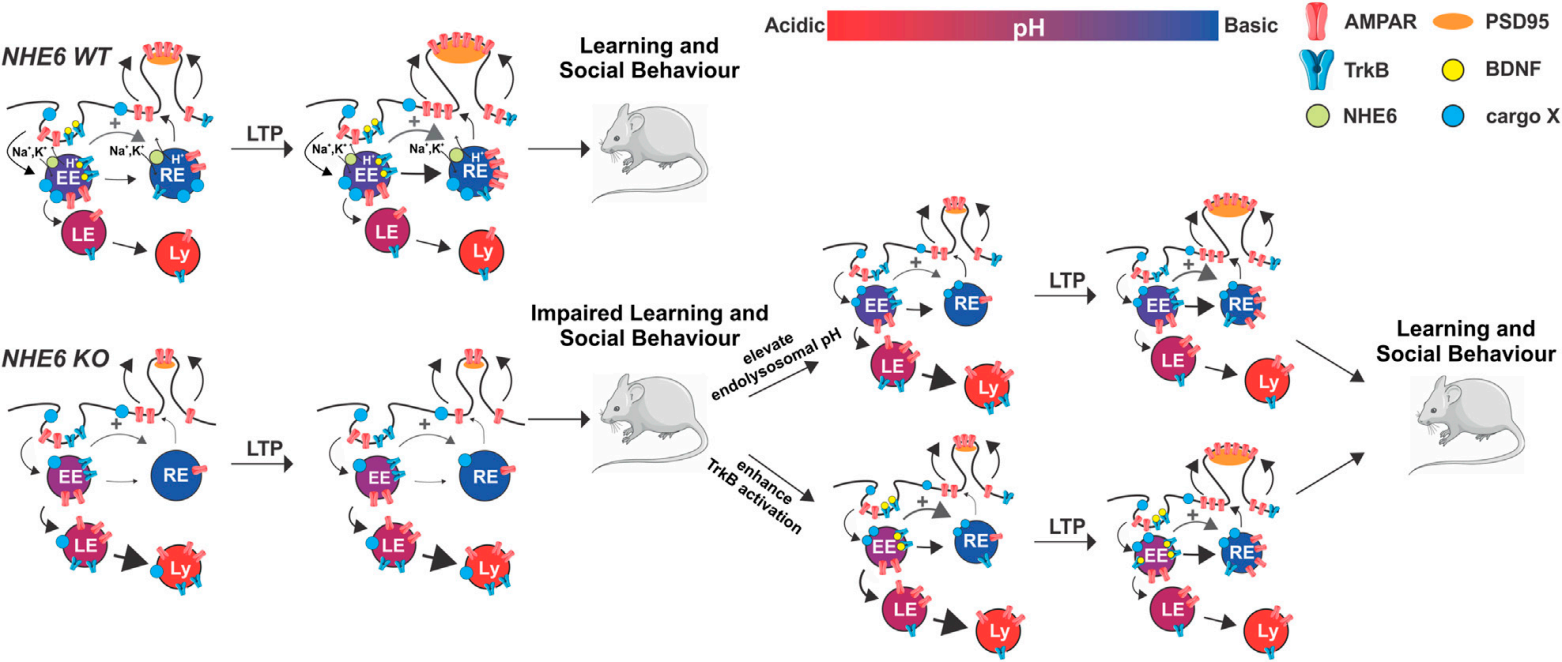
Schematic of impaired endosomal pH homeostasis and AMPAR and TrkB trafficking leading to defective synaptic potentiation and behavioural learning in NHE6 KO versus WT mice. Potential treatment strategies (i.e., elevate endolysosomal pH, enhance TrkB activation) to address these deficiencies in KO are indicated. Thickness of arrows represents relative extent of AMPA transport (black) or TrkB receptor signaling (gray). EE, early endosome; RE, recycling endosome; LE, late endosome; Ly, lysosome; BDNF, brain-derived neurotrophic factor; PSD95, postsynaptic density protein-95; cargo X, undefined membrane cargo.

Both NHE6 and NHE9 are critical during neurodevelopment. During murine embryogenesis, NHE6 is strongly present in developing fiber tracts across multiple brain areas, including the cortex, striatum, thalamus and hippocampus (Deane et al., 2013; Ouyang et al., 2013). NHE6 expression is then persistent throughout the postnatal brain, although it is significantly upregulated at postnatal day (PD) 50 during a period of intense synaptic pruning and refinement (Deane et al., 2013). In contrast, NHE9 expression is relatively low during development and begins to increase only postnatally, peaking at around PD 50 and declining thereafter (Ullman et al., 2018). Adult brain NHE9 expression appears to be restricted to the olfactory bulb, superficial cortical layers, and hippocampus (Lein et al., 2007). At the cellular level, NHE6 and NHE9 are broadly present within EEs and REs throughout the axons, somata, and spines of hippocampal pyramidal neurons (Deane et al., 2013; Ouyang et al., 2013; Ullman et al., 2018). These observations confirm the involvement of eNHEs in neuronal endosomes and suggest that they may function in long-range trafficking throughout neuronal compartments as well. Given that NHE6 is a critical mediator in the development of cellular polarity (Ohgaki et al., 2010), it is unsurprising that neurons deficient in functional NHE6 display reductions in axodendritic branching (Ouyang et al., 2013; Ilie et al., 2014; Ilie et al., 2016; Gao et al., 2019; Lizarraga et al., 2021). Accordingly, male *Nhe6* knock-out (KO) mice exhibit a pronounced overall undergrowth of the brain in addition to progressive volumetric losses in the cortex, striatum, hippocampus and cerebellum throughout adulthood (Xu et al., 2017). These reductions in neuronal branching and brain size are in accordance with the postnatal microcephaly commonly observed in human CS patients (Christianson et al., 1999; Pescosolido et al., 2014), which is highly suggestive of a reduction in adolescent brain development when NHE6 function is reduced or ablated. This has been hypothesized to arise from reduced activity of brain-derived neurotrophic factor (BDNF) (Ouyang et al., 2013), a critical neurotrophin that activates intracellular pathways regulating neuronal outgrowth, differentiation, plasticity and survival (Huang and Reichardt, 2001; Chao, 2003). Typically, binding of BDNF to its high affinity receptor tyrosine receptor kinase B (TrkB) results in the dimerization, autophosphorylation and endocytosis of the phosphorylated TrkB complex into signaling endosomes (SEs) (Xu et al., 2000; Rajagopal et al., 2004; Rex et al., 2007; Koleske, 2013). These SEs are then capable of activating downstream signaling cascades locally near the site of endocytosis to regulate neurite branching and elaboration, or undergo long-range retrograde transport back to the cell soma to activate transcriptional programs in the nucleus (Harrington and Ginty, 2013; Cosker and Segal, 2014). Importantly, this sorting decision can be influenced by the internal acidity of these SEs (Harrington et al., 2011), again underscoring the critical role of proper endosomal pH regulation. In hippocampal neurons, NHE6 colocalizes strongly with TrkB, suggesting that NHE6 is indeed involved in regulating the luminal pH of TrkB-containing SEs. Moreover, phosphorylated TrkB levels are reduced in *Nhe6* KO neurons (Ouyang et al., 2013) (Figure 3). These results engendered the hypothesis that the overacidification of endosomal pH induced by a lack of NHE6 function may lead to the mistargeting of TrkB to more acidic lysosomes to be degraded by resident hydrolases. Indeed, application of exogenous BDNF was sufficient to restore phosphorylated TrkB levels and neurite branching in cultured hippocampal KO neurons (Ouyang et al., 2013), implying that reversing the attenuation in BDNF/TrkB signaling was the root cause of these morphological impairments in neurons grown *in vitro*. Given that BDNF/TrkB signaling can also influence synaptic function and plasticity (Leal et al., 2017), it is conceivable that excitatory neurotransmission may also be impacted in murine *Nhe6* KO hippocampal neurons. Augmenting signaling through this pathway using specific TrkB agonists may thus prove to be beneficial in alleviating neurological deficits in CS patients harbouring *SLC9A6* mutations, as hippocampal BDNF/TrkB signaling is likely to be similarly diminished in these individuals.

How does the loss of eNHE function impact animal behaviour? Homozygous male *Nhe6* KO (*Slc9a6* ^*-/Y*^) mice exhibit several neurobehavioural abnormalities that resemble clinical phenotypes seen in CS patients. Perhaps the most evident are deficits in locomotor control, as revealed by their relatively poor performance in rotarod and balance beam tasks when compared to age- and sex-matched wild-type (WT) mice (Stromme et al., 2011). These mice further exhibit hyperactivity and anxiety-type behaviours in open field paradigms (Stromme et al., 2011; Sikora et al., 2016), as well as impaired visuospatial learning (Sikora et al., 2016) and diminished sensitivity to pain and pressure stimuli (Kerner-Rossi et al., 2019; Petitjean et al., 2020). Interestingly, female heterozygous mice (*Slc9a6* ^*+/−*^) show an intermediate phenotype compared to homozygous KO males (*Slc9a6* ^*-/Y*^) and WT male and female mice (Sikora et al., 2016), mirroring clinical findings in female carriers of deleterious *SLC9A6* alleles (Christianson et al., 1999; Pescosolido et al., 2019; Nan et al., 2022). These observations in heterozygotes are suggestive of a gene dosage effect of *SLC9A6* mutations upon these phenotypes, as the extent (or skewing) of X-inactivation (Belmont, 1996; Plenge et al., 2002) of either the WT or mutant *SLC9A6* allele in females likely accounts for the wide heterogeneity of their traits. Conversely, *Slc9a9/Nhe9* KO mice do not show obvious changes in gross hippocampal morphology or impairments in locomotion, anxiety, smell, or pain sensitivity (Yang et al., 2016; Ullman et al., 2018). However, they do exhibit classic characteristics of ASD, including repetitive behaviours and impaired social and olfactory function (Yang et al., 2016; Ullman et al., 2018). These differences allude to an intriguing divergence in how the loss of either eNHE impacts brain function in mammals and reflects the differing phenotypes observed in human patients harbouring genetic alterations in either NHE6 or NHE9.

## Roles of Endosomal NHEs in Excitatory Synaptic Function

These behavioural findings clearly establish the importance of eNHE function in the formation, function and plasticity of excitatory synapses. The precise roles of these eNHEs within the tripartite synapse (i.e., presynaptic and postsynaptic sites as well as neighbouring astrocytes) are uncertain, but recent studies have implicated their involvement in both pre- and post-synaptic vesicle function (Figure 1). In the presynaptic terminal, H^+^ import into synaptic vesicles (pH ∼5.7) is necessary for the loading of neurotransmitters (Miesenbock et al., 1998; Goh et al., 2011), underscoring the importance of vesicular pH regulation in this process. In the axons of cultured hippocampal neurons, signals of immunolabelled NHE6 and NHE9 endosomes overlap partially with synaptic vesicle 2 (SV2), a marker of presynaptic terminals (Ouyang et al., 2013; Ullman et al., 2018). NHE6 endosomes have also been observed in glutamatergic and GABAergic nerve terminals *via* mass spectrometry (Gronborg et al., 2010) as well as in purified glutamate-positive synaptic vesicles (Preobraschenski et al., 2014), where it may regulate vesicular cation transients during neurotransmitter loading. It is thus plausible that a reduction in NHE6 function could deleteriously impact quantal glutamate release. Indeed, fiber volleys recorded from area CA1 in *Nhe6* KO hippocampi are significantly reduced in amplitude when compared to WT. As these recordings are reflective of presynaptic firing and activation properties, this finding implies a possible loss in the number of overall functional synapses (Ouyang et al., 2013). However, WT and *Nhe6* KO do not significantly differ in the amplitude of paired-pulse ratio recordings at CA3-CA1 synapses (Ouyang et al., 2013), suggesting that the basal vesicular release properties of individual synapses may not be impacted by the loss of NHE6. Indeed, NHE6 has only been detected in a subset of synaptic boutons (Deane et al., 2013). Hence, a broad role for NHE6 in synaptic vesicle pH homeostasis and neurotransmitter uptake is unclear. Further investigations into the precise role of NHE6 within the presynaptic terminal are thus warranted to better understand the possible roles of this isoform in neurotransmitter loading and release.

By contrast, a recent study implicated NHE9 in presynaptic release. In contrast to studies in *Nhe6* KO, fiber volley amplitudes recorded from hippocampal area CA1 appeared to be comparable between WT and *Nhe9* KO, and thus the absence of NHE9 does not seemingly impact vesicular glutamate loading (Ullman et al., 2018). However, *Nhe9* KO CA3-CA1 synapses showed a significant increase in paired-pulse ratio, suggestive of a decrease in presynaptic release probability. This supposition was further supported by measurements showing that exocytosis of synaptic vesicles was impaired in *Nhe9* KO tissue due to a reduction in presynaptic Ca^2+^ influx. This was attributed to overacidification of endosomes in the absence of NHE9, as transient vesicle alkalinization using the V-ATPase inhibitor bafilomycin was sufficient to restore presynaptic Ca^2+^ entry in *Nhe9* KO neurons. This suggests that proper endosomal pH regulation in the axonal bouton is indeed a critical component in the trafficking or function of proteins regulating Ca^2+^ dynamics and neurotransmitter release. Further work into the involvement of NHE9 in regulating both neurotransmitter loading and presynaptic Ca^2+^ homeostasis will be necessary to elucidate its precise roles in axon boutons.

To date, the roles of NHE6 are perhaps better understood in relation to postsynaptic function. NHE6 accumulates at the base and head regions of dendritic spines and colocalizes with the excitatory postsynaptic scaffolding molecule PSD95 (Deane et al., 2013; Ouyang et al., 2013) (Figure 1). Furthermore, NHE6 colocalizes strongly with the GluA1 subunit of the AMPAR and is rapidly recruited to spines in response to NMDAR-mediated chemical LTP (Deane et al., 2013). Presumably, NHE6-mediated trafficking of AMPAR-containing vesicles is necessary for excitatory synaptic function and plasticity. As such, *Nhe6* KO CA1 pyramidal neurons demonstrated a marked reduction in overall dendritic spine density, with a loss of larger, more mature, dendritic spines concomitant with an increase in immature protrusions when compared to WT (Ouyang et al., 2013). These results strongly suggest that a complete loss of NHE6 function may reduce excitatory synaptic strength and activity-dependent remodeling, which has since been investigated with NHE6 mutants (discussed further below). In contrast, *Nhe9* KO neurons display comparable axodendritic branching and dendritic spine density to WT cells (Ullman et al., 2018). Nonetheless, electrophysiological recordings from *Nhe9* KO CA1 pyramidal neurons revealed cell-autonomous impairments of AMPAR and NMDAR function, suggesting that the loss of NHE9 can also alter glutamatergic neurotransmission in a pleiotropic manner (Ullman et al., 2018). Curiously, NHE9 does not appear to be involved in the trafficking of the AMPAR subunits, nor does the loss of NHE9 impact the surface expression of AMPARs or NMDARs (Ullman et al., 2018). Further insights into the role of NHE9 in mediating glutamatergic receptor trafficking and function are therefore necessary to better understand how changes in NHE9 function impact excitatory neurotransmission.

Both NHE6 and NHE9 are also expressed in astrocytes (Deane et al., 2013; Kondapalli et al., 2013; Ullman et al., 2018). With regards to NHE9, one report found that astrocytes lacking NHE9, or expressing ASD-associated variants in *SLC9A9*, exhibited a significant reduction in endosomal pH compared to those transfected with WT NHE9 (Kondapalli et al., 2013). Astrocytes expressing these ASD-associated *SLC9A9* variants also failed to upregulate the surface expression of excitatory amino acid transporter 1 (EAAT1), resulting in a decrease in glutamate uptake when compared to overexpression of WT NHE9 (Kondapalli et al., 2013). Altered glutamate clearance may thus underlie the aforementioned disturbances in AMPAR and NMDAR function in *Nhe9* KO neurons (Ullman et al., 2018), although direct assessments of excitatory transmission in cells expressing these NHE9 mutants have yet to be performed. Moreover, excess glutamate in the synaptic cleft likely imbalances the ratio of excitatory and inhibition transmission, which could account for the co-morbidity of epilepsy frequently observed in ASD (Kondapalli et al., 2014). Whether NHE6 also traffics EAAT1 in astrocytes is presently unclear, although magnetic resonance spectroscopy data of CS patients have shown an increase in glutamate concentration in the brain that may indicate enhanced excitatory transmission (Gilfillan et al., 2008; Schroer et al., 2010). Indeed, *Nhe6* KO mice do exhibit a pronounced increase in activated astrocytes and microglia within their gray matter (Xu et al., 2017; Kerner-Rossi et al., 2019). This observation may be a response to the heightened neurodegeneration detected in CS patients (Christianson et al., 1999; Gilfillan et al., 2008; Garbern et al., 2010; Schroer et al., 2010) and NHE6 KO mice (Stromme et al., 2011; Xu et al., 2017), but it could also be indicative of possible neuroinflammation which may not directly arise from its role in astrocytes. Interestingly, though not covered in this review, diminished NHE6 expression has also been detected in astrocytes associated with Alzheimer’s disease, suggesting a broader role for NHE6 is neurodegenerative pathophysiology (Prasad and Rao, 2018a; Prasad and Rao, 2018b). Taken together, it is evident that both NHE6 and NHE9 play very specific roles throughout the tripartite synapse, although important details remain to be discovered with regards to exactly which functions both eNHEs play within each compartment.

Tangentially, though not strictly considered an eNHE, the plasmalemmal NHE5 isoform also appears to play a role in postsynaptic remodeling and signaling. An intriguing study showed that in response to NMDAR-mediated chemical LTP, NHE5-containing vesicles are also recruited to, and undergo exocytosis at, dendritic spines. The addition of NHE5 at the postsynaptic membrane functions to extrude H^+^ into the synaptic cleft to negatively regulate NMDAR activation and restrict excessive spine expansion (Diering et al., 2011). Rapid recruitment of NHE5 from intracellular stores to the cell surface can thus serve to regulate the pH of synaptic microdomains, which can lend further precision to postsynaptic plasticity during LTP. Indeed, mice deficient in NHE5 displayed improved performance in learning and memory tasks accompanied by an increase in the number of hippocampal excitatory synapses in the hippocampus, which may have arisen from an augmentation in BDNF/TrkB signaling (Chen et al., 2017). Interestingly, past results have also shown that NHE5-mediated pH regulation may also be involved in the surface localization and retrograde signaling through the related tyrosine receptor kinase A (TrkA) (Diering et al., 2013). These data suggest that despite being canonically classified as a plasmalemmal NHE, activity-dependent trafficking of NHE5 to the synaptic cleft may serve to regulate neurotrophin signaling and plasticity. Additional work investigating the importance of NHE5 trafficking and pH regulation will undoubtedly reveal other intriguing roles of this isoform in synaptic physiology and regulation.

## Insights From Patient-Derived NHE6 Mutations

Given the monogenic nature of CS, recent work has also focused upon expressing CS-associated *SLC9A6* mutations in both non-neuronal cells and cultured neurons *in vitro* to investigate consequences to transporter activity and their impact on cell function. Most patient-derived variants harbor non-sense mutations that introduce a premature stop codon in the N-terminal transmembrane region of the protein, thereby preventing synthesis of a full-length protein and complete loss-of-function (LOF) (Gilfillan et al., 2008; Pescosolido et al., 2014). However, several CS-linked variants are missense mutations that result in production of a full-length protein, the effects of which are beginning to be elucidated (Roxrud et al., 2009; Ilie et al., 2014; Ilie et al., 2016; Gao et al., 2019; Ilie et al., 2019; Ilie et al., 2020).

In AP-1 cells [modified Chinese hamster ovary (CHO) cells containing negligible levels of NHE6 protein], most transfected NHE6 variants display impairments in post-translational oligosaccharide maturations and protein misfolding, resulting in enhanced ubiquitination and degradation by the proteasomal (ER-associated protein degradation, ERAD) (Meusser et al., 2005) and/or lysosomal (endosomal sorting complexes required for transport, ESCRT) pathways (Roxrud et al., 2009; Ilie et al., 2014; Ilie et al., 2016; Ilie et al., 2019; Ilie et al., 2020). Moreover, endosomes containing most of these NHE6 mutants are more acidic compared to those expressing NHE6 WT (with some exceptions, see below) (Ilie et al., 2016; Ilie et al., 2020), which is highly suggestive of impairments in endocytic processes. As such, expression of these mutant exchangers in HeLa cells and primary hippocampal neurons also disrupted transferrin uptake (Ilie et al., 2014; Ilie et al., 2016; Ilie et al., 2019; Ilie et al., 2020). These deficits appear to be cargo-selective, as uptake of epidermal growth factor was not disrupted in HeLa cells expressing an in-frame deletion mutant of NHE6 (p.Glu287_Ser288del; ΔES) (Ilie et al., 2016). It should be noted that although both HeLa cells and hippocampal neurons express endogenous NHE6, the introduction of exogenous NHE6 mutants exerts a dominant-negative effect as NHEs typically homodimerize to become functional. Thus, the expression of mutant NHE6 constructs results in the formation of WT and mutant NHE6 heterodimers that perturb the function of the WT fraction of NHE6 (Ilie et al., 2016). This effect is best exemplified in neurons, where transient expression of in-frame deletion mutants ΔES (Ilie et al., 2016) or ΔWST (p.Trp370_Ser_Thr372del) (Ilie et al., 2014) impaired neuronal branching, findings akin to those observed in NHE6 null neurons (Ouyang et al., 2013). Moreover, transfection of exchanger-deficient NHE6 variants into *Nhe6* KO neurons failed to rescue neuronal branching (Ouyang et al., 2013), indicating that ion exchange activity of NHE6 is necessary for neuronal morphogenesis. Furthermore, transient expression of some of these mutations into AP-1 cells or primary hippocampal neurons was sufficient to activate apoptotic cell death (Ilie et al., 2016; Ilie et al., 2020). These data strongly suggest that a lack of NHE6 function leads to the pronounced neurodegeneration observed in *Nhe6* KO mice (Stromme et al., 2011; Xu et al., 2017) as well as the progressive regression of symptoms in CS patients.

Additional evaluations of the ΔES mutation in cultured hippocampal neurons have recently revealed numerous interesting findings with regards to synaptic function. Neurons expressing ΔES displayed a significant reduction in the number of larger, more mature dendritic spines (Gao et al., 2019), again mirroring data from NHE6-deficient pyramidal neurons (Ouyang et al., 2013). Notably, the mutant exchanger was diverted away from EEs and REs towards LEs and lysosomes, which also resulted in a mistrafficking of the AMPAR GluA1 subunit to lysosomes. Consequently, ΔES-transfected neurons were unable to undergo structural and functional remodeling in response to NMDAR-dependent LTP. However, transiently inhibiting lysosomal function or V-ATPase activity was sufficient to partially restore these deficits in synaptic density and plasticity (Gao et al., 2019). These results suggest that NHE6-mediated pH regulation is necessary to mediate proper AMPAR recycling through the endosomal system, which allows AMPARs to be rapidly inserted into PSDs from intracellular stores during LTP. In the absence of functional NHE6, however, these vesicles become hyperacidified, resulting in the mistrafficking of AMPARs to lysosomes for subsequent proteolysis (Figure 3). This study provides some of the first data showing how excitatory postsynaptic trafficking and remodeling may be impaired when NHE6 function is downregulated. Importantly, it also suggests that reversing dysregulations in vesicular pH—for instance, by using Na^+^/H^+^ ionophores (e.g., monensin) that mimic NHE6 function (Mollenhauer et al., 1990; Prasad and Rao, 2015) or by employing protonation agents (e.g., chloroquine) that alkalinize endosomal pH (Akpovwa, 2016; Al-Bari, 2017)—may prove to be beneficial therapeutic strategies to treat CS patients (Figure 3). How the ablation of NHE6 function affects synaptic plasticity at the level of the whole circuit remains to be fully elucidated, although we predict that functional and structural LTP will be similarly impaired in *Nhe6* KO hippocampi.

Parenthetically, certain NHE6 mutants appear to diverge slightly from these patterns. An interesting example of this is the *de novo* missense NHE6 variant p.Gly218Arg (G218R). When expressed in both AP-1 cells and primary hippocampal neurons, vesicles containing G218R were more alkaline compared to controls (Ilie et al., 2019). This observation was suggestive of a gain-of-function effect, in contrast to the loss of function associated with the ΔWST and ΔES mutants. While a fraction of G218R was subjected to ERAD, the alkalinization of endosomal pH instead resulted in the trafficking of a significant portion of the mutant exchanger to LEs and extracellular release in exosomes upon LE fusion with the plasma membrane (Ilie et al., 2019). Nonetheless, neurons expressing G218R showed similar morphological impairments to those expressing LOF mutations, including reduced dendritic arborization and spine density (Ilie et al., 2019). These results underscore the importance of precisely regulating endosomal pH within strict physiological bounds, as dramatic shifts in either acidification or alkalinization can impose similarly disrupt neuronal and synaptic morphology. This is especially important to consider if endosomal alkalinization agents or genetic rescue approaches are to be used in the treatment of CS, as such therapies will therefore have to be tailored for each specific mutation. In contrast, other mutations identified in CS patients have been demonstrated to exert only negligible effects on exchanger properties and cellular physiology. Specifically, two additional missense mutations, one in the extreme N-terminal cytoplasmic segment (pAla9Ser; A9S) and another in the regulatory C-terminal cytoplasmic domain (Arg568Gln; R568Q) appear to have comparable biochemical characteristics (i.e., biochemical maturation, stability, and vesicular pH regulation) to that of WT NHE6 when expressed in AP-1 cells (Ilie et al., 2020). Engineering the equivalent A9S mutation in mice also revealed normal brain development and neuronal arborization and endosomal pH regulation (Ouyang et al., 2019). In addition, this NHE6 variant appears to be relatively common in healthy individuals (genome aggregation database, gnomAD; RRID:SCR_014964), confirming that this mutation alone does not likely lead to neurological impairments. Conversely, while the R568Q mutation also appeared to be relatively benign in heterologous cells, the effects of this particular variant upon synaptic transmission and plasticity in neurons have yet to be studied. These findings suggest that some patients harbouring NHE6 mutations may have been misdiagnosed, and additional genome-wide analyses will be required to elucidate the true cause of their pathophysiology.

In conclusion, although the prevalence of recorded mutations in both NHE6 and NHE9 continues to increase, treatment options are extremely limited for patients afflicted by neurodevelopmental disorders that arise from altered eNHE function. This paucity arises from an incomplete understanding of the complex roles these eNHEs play at the cellular, molecular and behavioural levels. Even though both NHE6 and NHE9 similarly localize to endosomal compartments, these eNHEs can nevertheless play divergent roles owing to the differences in their spatiotemporal expression patterns. Specifically, the current literature suggests that loss of NHE6 may be especially important during the formation, establishment and potentiation of excitatory synapses in the developing brain, whereas NHE9 may play a greater role in circuit refinement in the postnatal nervous system. This is further reflected in the reciprocity in their expression in ASDs (Schwede et al., 2013), which suggests that NHE9 hyperfunction may also be detrimental to nervous system refinement during adolescence. Moreover, the fact that mutations in NHE6 appear to be much more deleterious than those in NHE9 suggests that the former indeed plays a greater role in excitatory synaptic formation and plasticity, although the contributions of NHE9 within presynaptic terminals and astrocytes should not be discounted. While the growing interest in these exchangers and, more broadly, the role of endosomal dynamics in synaptic function is encouraging, there clearly remains an urgent need to further understand the unique roles of eNHEs in the tripartite synapse. Such knowledge will help guide development of translational strategies to ameliorate the broad range of neurological impairments associated with mutations in these genes.
